# Construction costs and tradeoffs in carnivorous pitcher plant leaves: towards a pitcher leaf economics spectrum

**DOI:** 10.1093/aob/mcaf024

**Published:** 2025-04-12

**Authors:** Kadeem J Gilbert, David Armitage, Ulrike Bauer, Kenji Fukushima, Laurence Gaume, Rachel Love, Qianshi Lin, Sukuan Liu, Sylvie Martin-Eberhardt, Jonathan Millett, Tanya Renner, Mathias Scharmann, Chris Thorogood

**Affiliations:** W.K. Kellogg Biological Station, Department of Plant Biology, and Program in Ecology, Evolution & Behavior, Michigan State University, Hickory Corners, MI, USA; Integrative Community Ecology Unit, Okinawa Institute of Science and Technology Graduate University, 1919-1 Tancha, Onna-son, Kunigami-gun, Okinawa, Japan; Department of Biosciences, University of Exeter, Geoffrey Pope Building, Stocker Road, Exeter, UK; Center for Frontier Research, National Institute of Genetics, 1111 Yata, Mishima, Shizuoka 411-8540, Japan; AMAP, Montpellier University, CNRS, CIRAD, INRAE, IRD, Montpellier, France; Learning Commons, Kalamazoo College, 1200 Academy Street, Kalamazoo, MI, USA; Department of Entomology, The Pennsylvania State University, 501 Agricultural Sciences and Industries Building, University Park, PA, USA; Department of Biology, Colorado State University, 251 W Pitkin St, Fort Collins, CO 80521, USA; Department of Ecology and Evolutionary Biology, University of Colorado Boulder, 1900 Pleasant Street, Boulder, CO 80309, USA; W.K. Kellogg Biological Station, Department of Plant Biology, and Program in Ecology, Evolution & Behavior, Michigan State University, Hickory Corners, MI, USA; Department of Geography & Environment, Loughborough University, Epinal Way, Loughborough, LE2 1YE, Leicestershire, UK; Department of Entomology, The Pennsylvania State University, 501 Agricultural Sciences and Industries Building, University Park, PA, USA; Institute for Biochemistry and Biology, Genetics Research Group, University of Potsdam, Building 26, Room 1.50, Karl-Liebknecht-Str. 24-25, 14476 Potsdam, Germany; Botanic Garden, University of Oxford, Rose Lane, Oxford, OX1 4AZ, UK; Department of Biology, University of Oxford, Zoology Research and Administration Building, 11a Mansfield Rd, Oxford OX1 3SZ, UK

**Keywords:** Carnivorous plants, pitcher plants, leaf economic spectrum, *Cephalotus*, *Nepenthes*, *Sarracenia*, *Heliamphora*, *Darlingtonia*

## Abstract

**Background:**

Leaf economics theory holds that physiological constraints to photosynthesis have a role in the coordinated evolution of multiple leaf traits, an idea that can be extended to carnivorous plants occupying a particular trait space that is constrained by key costs and benefits. Pitcher traps are modified leaves that may face steep photosynthetic costs: a high-volume, three-dimensional tubular structure may be less efficient than a flat lamina. While past research has investigated the photosynthetic costs of pitchers, the exact suite of constraints shaping pitcher trait variation remain under-explored, including constraints to carnivorous function.

**Scope:**

In this review, we describe various constraints arising from the dual photosynthetic and carnivorous functions of pitchers arising from developmental, functional, budgetary and environmental factors. In addition, we identify the data required to establish the leaf economics spectrum (LES) for carnivorous pitcher plants (CPPs), and – owing to the multifunctional roles of pitcher leaves – discuss difficulties in placing pitchers onto existing frameworks.

**Conclusion:**

Because pitcher traps serve multiple functions, both photosynthesis and nutrient acquisition (carnivory), they are difficult to place in the context of the LES, especially in light of a current lack of trait data. We describe a spectrum across the independent CPP lineages in approaches to balancing carnivory–photosynthesis tradeoffs. Future efforts to collect relevant data can clarify the forces that shape observed pitcher trait variation, and increase understanding of principles that may be ultimately generalized to other plants.

## INTRODUCTION

The leaf economics spectrum (LES) is a conceptual framework that has advanced knowledge of the correlations among leaf traits, clarified environmental correlates of these traits, and revealed tradeoffs in leaf construction by way of constraints over nutrient and energy allocation (Wright *et al.*, 2004). This framework focuses on a few key functional traits that can be measured in a standardized way across the global diversity of leaves. Doing so de-emphasizes the idiosyncrasies of specific lineages to identify the general factors constraining the geographical, environmental and phylogenetic distributions of traits. For instance, shorter-lived leaves tend to invest highly in photosynthesis and nutrient acquisition at the expense of leaf mass per unit area (LMA), in contrast to longer-lived leaves. This tradeoff is predicted to be because the balance of the costs and benefits of increased LMA has a longer payback-time than a short leaf lifespan allows. Because leaves are costly in terms of nutrient use, the key environmental controls over variability are found across light and nutrient gradients. In sunny conditions, thicker leaves with higher nitrogen concentrations are more effective at intercepting light; shade leaves tend to be thinner with lower nitrogen concentrations. In nutrient-replete conditions, short-lived leaves with low LMA and high N concentrations are more effective at fast growth in a competitive environment; in nutrient-poor conditions, long-lived leaves with high LMA and low N concentrations use the limited nutrient resource more conservatively. The utility of this trait-based framework has been extended in consideration of other tissues and organs as well, including wood (Chave *et al.*, 2009), roots (de la Riva *et al.*, 2021) and flowers (Roddy *et al.*, 2021). One major knowledge gap concerns the tradeoffs involved in multi-function plant structures, such as those required for both carbon fixation and nutrient or water acquisition, for example the photosynthetic roots of epiphytic orchids and the prey-trapping structures of carnivorous plants (CPs) – the latter of which are the focus of this review.

Plant carnivory is a habit defined by multiple components, including adaptations for the attraction, capture, retention, digestion and assimilation of nutrients derived from animal prey. Carnivorous plants have evolved multiple times independently: at least ten origins within Angiosperms, over 800 total species (Ellison and Adamec, 2018). This has been linked to the selective pressures of nutrient-limited environments (Givnish *et al.*, 1984). The leaves of this group of plants function as traps for catching and digesting arthropods, but the traits of carnivorous leaves are themselves very variable, comprising multiple distinct trapping strategies including snap traps, sticky traps and pitcher traps, among others (Ellison and Adamec, 2018). The need to function as a trap means that CP leaves may fall outside the range of variation for more ‘typical’ leaves, especially in pitcher plants. Additionally, it is interesting to contemplate why carnivory overall comprises such a wide variety of strategies, while other plant functions may be relatively more constrained. Considering specifically pitfall traps, this strategy is relatively rare amongst land plants [~200 pitcher plant species, ~837 CPs in total (Ellison and Adamec, 2018); see Box 1], and species employing this trait tend to be patchily distributed and most abundant in remote regions that can be difficult to access. Furthermore, it is often unclear how some canonical plant leaf traits are measured on such complex, 3-D structures. For these reasons, pitcher plants are not commonly included in large-scale trait-based studies (Box 2), yet the atypical traits of pitchers could serve as a key for a more comprehensive, global view of the LES.

There are many reasons why pitcher plants represent an interesting extension of the LES. As the currency of the LES is carbon produced by photosynthesis, leaves tend to be relatively flat because a high surface-to-volume ratio is the most efficient form for photon capture. Thus, the high-volume, 3-D, tubular leaf structure of pitcher plants represents an extreme deviation from photosynthetic efficiency. Therefore, a first key question is whether the LES model is suitable for pitcher leaves to begin with. Moreover, pitcher plants are of interest because they separate photosynthesis and nutrient acquisition both temporally and spatially: for example, *Cephalotus* leaves shift between these functions through time (depending on conditions), while *Nepenthes* leaves comprise a 3-D pitcher for prey capture, together with a 2-D lamina for photosynthesis, as we discuss below. Beyond their 3-D architecture, pitchers are also multi-role organs (though this role may be reduced in *Nepenthes* pitchers) that must capture light and also function as ‘above-ground roots’ to acquire growth-limiting nutrients such as nitrogen and phosphorus. This extreme alteration of the photosynthetic structure may also explain the widely reported gene loss in their plastid genomes, perhaps suggesting a downsizing of photosynthetic function at a molecular level (Wicke *et al.*, 2014; Ross *et al.*, 2016; Fu *et al.*, 2023; Silva *et al.*, 2023). From what has been measured thus far, pitcher plants tend to have low photosynthetic rates (*A*_N_), and may have atypically high rates of dark respiration (*R*) due to their unusually high levels of alternative oxidase (Pavlovič and Kocáb, 2022). An in-depth exploration of pitcher traits considering the particular set of functions the plants must achieve can reveal novel ways of applying the LES, or even expand our view of the broader total trait space onto which leaves can fall. At one level, the LES can be a tool to understand the vast variation in pitcher size and morphology observed in nature; at another level, incorporating pitchers into the LES opens up the opportunity to test the general validity of the LES for other highly modified leaves (succulent leaves, tendrils, spines, etc.).

The LES is, by definition, a framework for understanding covariances between leaf traits and relating these covariances to developmental, functional, ecological or evolutionary factors. To provide insight on pitcher trait space, we have chosen to focus on four potential constraints to the evolution of trait variance within this group: (1) developmental constraints, i.e. the mechanisms responsible for the production of pitcher plant morphologies; (2) functional constraints, i.e. the adaptive ends to variation in these structures/traits; (3) budgetary constraints, i.e. constraints due to a major functional tradeoff observed in this group and differential allocation to either function; and (4) environmental constraints, i.e. how abiotic factors may further shape their trait distribution. These four angles represent major axes that can be applied to diverse plant groups. Further, we specifically investigate each constraint in relation to two major functional categories pitcher leaves must achieve: photosynthetic and carnivorous functions. Recognition of the specific importance of both of these functional categories (and the tradeoffs between them) to pitcher plants is a major motivator for exploring this proposed framework. In the following sections, we present evidence – where available – and interpretations on the existence of trait variation and potential covariances or tradeoffs at each of these biological constraints. In a sense, these constraints can be envisioned as a series of filters from what is theoretically possible to that which is evolutionarily feasible and phenotypically realized. In considering which traits to measure and the potential factors constraining them, we also outline important, unanswered questions that only coordinated, trait-based approaches can address, which we hope will motivate additional research on pitcher plant traits and enable an LES analogue for these multi-function plant structures.

## DEVELOPMENTAL CONSTRAINTS ON PHOTOSYNTHESIS

Carnivorous plants face the unique challenge of balancing two primary functions within a single organ: photosynthesis and carnivory. As a response, some lineages, such as *Nepenthes*, spatially partition these functions: the distal portion of the leaf in *Nepenthes* forms a pitcher to capture prey, while the proximal portion is flattened and oriented optimally for photosynthesis. This division utilizes the inherent segmentation found in most flowering plant leaves, which typically consist of three parts: the leaf blade, the petiole and the leaf base (Franck, 1976). In pitcher plants, either the leaf blade itself develops into a trap, or traps emerge from apical tendrils extending from the blade. In some *Nepenthes* species, trapping pitchers have almost no photosynthetic activity, indicating a high degree of functional compartmentalization and specialization (Pavlovič *et al.*, 2007; Capó-Bauçà *et al.*, 2020). The tendril in this genus can also have an important climbing and support function. The leaf base, including stipules, can contribute to photosynthesis in some flowering plant lineages and forms the flat, photosynthetic portion of *Nepenthes* leaves.

While *Nepenthes* uses a compartmentalized leaf organization, *Cephalotus* is phenotypically plastic, showing a shift in leaf type depending on environmental conditions. For example, in a controlled environment, *Cephalotus* generates photosynthetic leaves at 15 °C and carnivorous leaves at 25 °C. This shift aligns with the seasonal temperature variations of natural habitats and possibly correlates with the likelihood of capturing prey. Occasionally, these plants may produce leaves that are neither fully photosynthetic nor fully carnivorous (Fukushima *et al.*, 2021), adding another layer of complexity to the adaptive benefits and costs of leaf development. *Sarracenia* utilizes a hybrid approach, combining leaf segmentation with phenotypic plasticity to optimize both photosynthesis and carnivory (Ellison and Gotelli, 2002).

## DEVELOPMENTAL CONSTRAINTS ON CARNIVORY

Once leaves have evolved to form pitcher traps, there are still a number of structural or developmental factors constraining their overall morphological breadth. In *Heliamphora*, the morphological evolution of adult pitchers was found to be structurally constrained, as indicated by the strong evolutionary correlations between pitcher shape variations (i.e. pitcher stoutness and curvature) and maximum pitcher size (length) (Liu and Smith, 2023). *Heliamphora* species with slender or straight pitcher shapes are associated with larger pitcher sizes, while taxa with stout or curved pitcher morphologies correlate with smaller pitcher sizes. Liu and Smith (2023) postulated that stout or curvy pitchers are less structurally stable compared to slender or straight pitchers because the centre of their pitcher mass is further away from the growing point on the rosette, which provides structural support.

Sticky trichomes have repeatedly evolved across multiple plant lineages, and thus it may be simple to envision the development and evolution of the sticky trap type from non-CPs – though Kaplan and Specht (2020) bring the identity of sundew tentacles as trichomes into question and proposed, with support from early observations made by Diels (1906), that the tentacles are homologous with pinna lobes. In contrast to sticky traps, the snap trap has only evolved exactly once (Dionaea + Aldrovanda clade), and has evoked a more complex hypothesis for its stepwise evolution from non-CP leaf to sticky trap to snap trap (Gibson and Waller, 2009). Likewise, one might imagine pitchers to be developmentally complex. In contrast to the expectation that drastic genetic changes would be needed for transforming a flat leaf into a pitcher, studies on the development of pitcher leaves in *Sarracenia* (Fukushima *et al.*, 2015) and the formation of follicle-shaped traps in the carnivorous bladderwort genus *Utricularia* (Whitewoods *et al.*, 2020) indicate that a pitcher-shaped leaf may have evolved through relatively simple genetic alterations. These genetic alterations specifically modify the adaxial–abaxial patterning during early leaf development. In the case of *Sarracenia*, this modification in tissue patterning is achieved through a distinct pattern of cell division (Fukushima *et al.*, 2015). While the formation of a pitcher shape is a crucial step, the existence of a fluid-holding pitcher structure alone is insufficient for successful nutrient capture. Instead, the trap leaves must also invest in materials for prey attraction, retention, digestion and nutrient absorption. This involves a suite of complex morphological traits including localized hypertrophy leading to the development of remarkably variable and often specialized peristomes and opercula (Arber, 1941). Thus, physical limits to vascular packing and resource provisioning during pitcher development may constrain the size and complexity of the leaves. Nevertheless, the realization that modifying the basic leaf structure is less complicated than previously assumed offers new perspectives. It suggests that the primary challenges in the evolution of pitcher leaves probably lie in areas other than simply altering the overall leaf shape, and hence there is a need for further study on the bioenergetics of pitcher leaf development, which arises from tradeoffs in nutrient and energy capture.

## FUNCTIONAL CONSTRAINTS ON PHOTOSYNTHESIS

Most CP leaves, and all pitcher traps, must solve the balancing act of photosynthetic functioning and nutrient acquisition. As shown by the cost/benefit model of carnivory, the photosynthetic costs of carnivorous leaves may not pay off if the environment is also limited in water and light (Givnish *et al.*, 1984). Pitcher traps, like other CP leaves, have greatly diminished photosynthetic rates and nutrient use efficiency (PNUE) compared to typical plants (Ellison, 2006); trap photosynthetic efficiency increases when they are fed (Pavlovič *et al.*, 2009; Pavlovič and Saganová, 2015). It is currently unclear whether pitchers are even more inefficient than other types of trap leaves (e.g. sticky traps), given limited sampling, but there are reasons to suspect they could be on the lower end of the range within the generally low photosynthetic rates for unfed CPs. Considering the role of specific leaf traits, photosynthesis is hypothetically most effective when a leaf is oriented horizontally, i.e. perpendicular to the sun; however, for prey retention in a pitfall trap, vertical trap walls are most effective. In a sufficiently warm, bright and humid environment, the rate of photosynthesis is directly proportional to the chlorophyll content of a leaf. In other words, greener leaves may be more efficient at photosynthesizing. On the other hand, greenness may or may not be an advantage to pitcher traps as contrasting colours such as red and yellow are hypothesized to function as attractive signals, as in flowers (Juniper *et al.*, 1989); however, evidence for the functional role of red pigments in carnivorous trap leaves is equivocal (Schaefer and Ruxton, 2008; Bennett and Ellison, 2009; Foot *et al.*, 2014; Millett *et al.*, 2017; Annis *et al.*, 2018; Gilbert *et al.*, 2018; Martin-Eberhardt *et al.* 2025). Pigmentation may, for example, rather be necessary for photoprotection in open, sunny habitats. Red pigmentation may correlate strongly with abiotic (light) gradients at a global scale for sundews (Millett *et al.*, 2017), while this may or may not be the case for any of the multiple lineages of pitcher plants. Regardless, pitcher traps are apparently not constrained by a need for photosynthetic pigments. This is most strikingly apparent in species with white or translucent patches on their pitchers. This reduced investment in chlorophyll relative to other pigments (or lack thereof) may explain why pitcher leaves tend to have especially low photosynthetic rates for their LMA, relative to other plants, perhaps implying that the additional demands on leaves (necessitating a particular LMA) constrains photosynthetic rates (*A*_max_) (Farnsworth and Ellison, 2008). Thus, the demands of photosynthetic and carnivorous functions may not necessarily be aligned, which in turn might be the reason for pitcher plants residing on the margins of many leaf trait scaling relationships such as the negative association between *A*_max_ and LMA.

Relatively few studies have measured a suite of traits of fundamental interest to photosynthetic capacity, such as stomatal traits and leaf thickness (Paluvi and Mukarlina, 2015; Mansur, 2017; Osunkoya and Muntassir, 2017; Meriko and Abizar, 2017; Ghazalli *et al.*, 2019; Huda *et al.*, 2022). Paluvi and Mukarlina (2015) observed intraspecific changes in *Nepenthes* leaf anatomy with light environment consistent with general expectation, namely *N. gracilis* had thinner epidermal leaf thickness in shade compared to open habitat. Of note, the results of this anatomical study were not just limited to the leafy lamina, but held true for the tendrils as well. In addition to leaf thickness, stomatal density also differed, with lower stomatal counts for shade plants. Meriko and Abizar, (2017) looked more specifically at stomatal trait differences across three *Nepenthes* species, *N. gracilis*, *N. reinwardtiana* and *N. ampullaria*. While *N. ampullaria* was classified as being of the anomocytic type, the other two species were classed as the less common actinocytic type with radially arranged subsidiary cells. *Nepenthes reinwardtiana* stood out among the other two species in having a lower stomatal density (~115 per mm^2^ compared to 344 and 323 per mm^2^ in *N. ampullaria* and *N. gracilis*, respectively) and larger stomatal size (11 776 μm^2^ vs. 7360 and 8555 μm^2^ in *N. ampullaria* and *N. gracilis*, respectively). It would be fruitful to combine anatomical studies with physiological data to better understand interspecific variation in photosynthetic efficiency. For instance, Mansur (2017) investigated CO_2_ absorption rates in 15 *Nepenthes* species and hybrids. *Nepenthes reinwardtiana* and *N. gracilis* were among these as having amongst the highest CO_2_ absorption rates (9.30 and 9.71 μmol m^−2^ s^−1^, respectively); *N. ampullaria* was observed to have the lowest rate (3.74 μmol m^−2^ s^−1^). As it stands, anatomical data are too sparse to draw conclusions on whether stomatal type, size, density or other traits impact CO_2_ absorption potential. Mesophyll conductance was found to be an important constraint to photosynthetic efficiency in *Nepenthes* (Capó-Bauçà *et al.*, 2020), and thus more research on leaf anatomy should prove fruitful to this end.

**Fig. 1.**
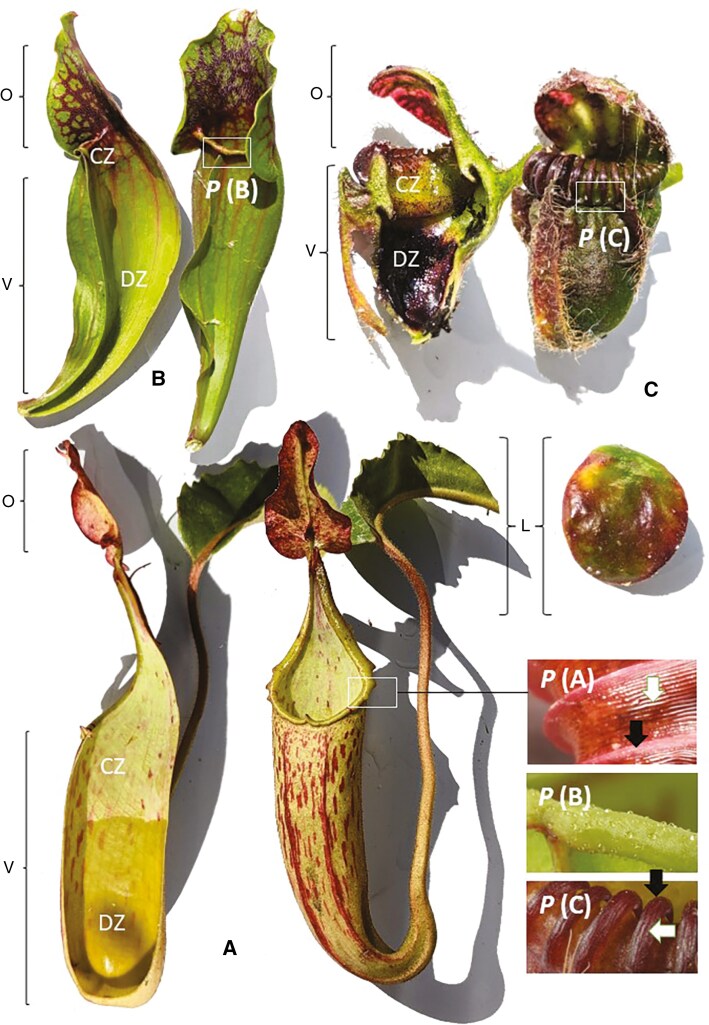
The convergent pitcher structures and surfaces of: (A) *Nepenthes*, (B) *Sarracenia* and (C) *Cephalotus.* DZ = digestive zone; L = photosynthetic (non-tubular) lamina of *Nepenthes* (left) and *Cephalotus* (right: note the lamina is not attached to the pitcher); CZ = conductive zone; V = vessel; O = operculum; P = peristome (followed by the letter denoting the genus, as above); bottom right boxes show magnification of peristomes; note the primary ribs (black arrow) and secondary ribs (white arrow) present in *Nepenthes* and *Cephalotus* which are absent in *Sarracenia. Photo credit: C. Thorogood*

The three major lineages of pitcher plants (Cephalotaceae, Nepenthaceae and Sarraceniaceae) mitigate photosynthetic functional tradeoffs differently (Fig. 1). Species in the Sarraceniaceae rely on a high degree of phenotypic plasticity in pitcher morphology throughout growth and development to separate these two functions temporally. *Darlingtonia* and *Heliamphora* produce juvenile pitchers upon germination that are morphologically distinct (i.e. in pitcher size and shape) from those in adult form. These two leaf forms may show divergent tradeoff strategies between photosynthetic cost and prey capture during development. In *Sarracenia*, certain environmental conditions favour the conversion of the entire leaf into a flat keel, a structure known as a ‘phyllodium’. Many species produce phyllodia in late summer when conditions become less favourable for carnivory (Schnell, 1980; Beaulac *et al.*, 2002; Ellison and Gotelli, 2002) or generally in response to excess nitrogen deposition in the environment, which obviates the benefit of prey capture (Ellison and Gotelli, 2002). Similarly, the pitchers of *Heliamphora* growing in light-limited conditions produce pitchers with reduced carnivorous traits (i.e. elongated pitcher bodies, reduced funnel volume, loss of pigmentation, and nectar production on the pitcher and nectar spoon) as a temporal tradeoff for photosynthesis (McPherson *et al.*, 2011). Likewise, the ratio of anthocyanin to chlorophyll in the forked ‘tongue’ appendage in *Darlingtonia* decreases with sun exposure, indicating that leaves of this species can plastically respond to light availability with no apparent consequences for prey capture success (Armitage, 2016*a*). For all these examples, a case can be made for considering the photosynthetic and the trapping leaf phases/parts separately when considering their place in the LES. This is corroborated by the fact that both can have vastly different life spans. Because leaf senescence in pitcher leaves generally begins at the distal end of a leaf, in *Nepenthes*, this results in the photosynthetic lamina outliving its associated trap by months (or possibly years if the period of time prior to trap production is factored in). In Sarraceniaceae leaves, however, the first region to senesce is also the most active region for light capture, but the digestive zone at the shaded base of the leaf can remain alive and function well into a second growing season.

## FUNCTIONAL CONSTRAINTS ON CARNIVORY

While the traditional LES is largely centred on the photosynthetic function of leaves, nutrient acquisition is also a key function of pitcher traps (i.e. carnivory). In this way, the functional analogue for carnivorous pitchers may contain properties of both roots and photosynthetic leaves. Carnivory requires a specific suite of traits, which may have unique sets of costs and tradeoffs distinct from those associated with photosynthesis. Pitchers generally have extrafloral nectaries that attract prey with a sugar reward (i.e. a direct cost in carbon paid to carnivory). Some pitcher plants attract prey through the production of volatile organic compounds (VOCs) (Jaffe *et al.*, 1992; Di Giusto *et al.*, 2008; Jürgens *et al.*, 2009; Ho *et al.*, 2016; Hatcher *et al.*, 2020; Dupont *et al.*, 2023), nectar (Merbach *et al.*, 2001; Bennett and Ellison, 2009) and/or possibly through reflectance patterns (Joel *et al.*, 1985; Moran *et al.*, 1999; Moran *et al.*, 2012), which may all have costs in terms of secondary metabolism. Constraints may be considered in concert or separately across any of the individual functional components of carnivory (attraction, capture, retention, digestion and assimilation of prey nutrients).

While some data support the hypothesized attractive functions of the traits listed above, more experimental work is generally needed to confirm the role of specific traits as signals to specific prey. In contrast to the other four functional components, elucidating animal behaviour is just as critical to empirically probing attractive function as is knowledge from the perspective of the plant. Capture and retention may also involve some elements of animal behaviour, but digestion and assimilation can be broadly understood from plant physiology alone. One complex trapping strategy with strong empirical support from a behavioural experiment is the ‘light-trapping’ strategy found in *Nepenthes aristolochioides*. This species produces pitchers with a dome-shaped roof and a near-vertically oriented (lateral) opening; the roof has extensive white/translucent patterns (‘windows’ or ‘fenestrations’). Moran *et al.* (2012) demonstrated that flies (*Drosophila melanogaster*) are attracted to the bright entrance, become disoriented by the false exit of light shining through the back and therefore become trapped. Various pitcher plant species across multiple genera possess such fenestrations, including *Nepenthes klossii*, *Sarracenia minor*, *S. psittacina* and *Darlingtonia californica*. More work is needed to confirm the light-trapping strategy in these other species [but see Schaefer and Ruxton (2014) for *S. minor*]; further, the effectiveness of this strategy on a broader range of insect species remains to be tested.

In contrast to attraction, traits involved in capture and retention are generally better understood on a fundamental, mechanistic level. Most *Nepenthes* possess a collar-shaped pitcher rim (peristome) that turns slippery when wet to capture insects via aquaplaning (Bohn and Federle, 2004; Bauer *et al.*, 2008; Bonhomme *et al.*, 2011b; Lessware *et al.*, 2025). Approximately two-thirds of *Nepenthes* species additionally produce a wax crystal coating on the inner pitcher wall that prevents insect attachment and favours both insects falling into the trap (Juniper *et al.*, 1989; Gaume *et al.*, 2002) and retention of captured prey (Juniper *et al.*, 1989; Gaume *et al.*, 2002, 2004; Di Giusto *et al.*, 2009; Bauer *et al.*, 2012*a*). The relative length of the waxy zone varies from species to species (Di Giusto *et al.*, 2009; Benz *et al.*, 2012), and a negative correlation has been found between the relative length of the waxy zone and the relative width of the peristome (Benz *et al.*, 2012), suggesting an investment tradeoff for the plants between these two types of slippery structure. Two *Nepenthes* species (*N. gracilis* and *N. pervillei*) are known to produce two distinct forms of wax plates on the pitcher wall and underside of the lid, which function differently, as part of a complex trapping strategy (Bauer *et al.*, 2012*b*; Chomicki *et al.*, 2024). In this case, ants are able to crawl on the lid underside and only become dislodged when the lid is struck by raindrops in this ‘spring-board’ trapping strategy; this involves a particular wax platelet morphology and biomechanical modifications to the lid. Interestingly, the springing action of some lids is not a consequence of the springing lids being made of a different material than non-springing lids, but rather relies on *how* that tissue material is arranged (Chomicki *et al.*, 2024). Sarraceniaceae generally have a less prominent waxy layer, but have downward-pointing slippery trichomes (Bauer *et al.*, 2013); however, a few species such as *S. leucophylla*, *S. alata*, *S. flava* and *S. rubra* do have notable wax crystals as well (Poppinga *et al.*, 2010). Pitcher fluid may also be involved in prey capture and retention, as prey drown in the fluid and can be harmed directly by the acidic conditions (Bazile *et al.*, 2015). Pitcher fluid is largely plant-produced (especially in *Nepenthes*), and thus fluid production could hypothetically incur a cost when water is limiting. Pitcher plants, like other CPs, are presumed to be solely adapted to conditions that are not water-limited (Givnish *et al.*, 1984), but some *Nepenthes* species indeed occur in seasonally dry areas (McPherson *et al.*, 2009), so it may be interesting to investigate whether fluid production traits (i.e. volume produced) may vary in these. Some pitcher plant species produce little of their own fluid and instead rely largely on filling up with rainwater; this includes *Sarracenia purpurea*, *Nepenthes ampullaria* and many *Heliamphora* spp. These species appear to invest less in fluid production, but do not occur in dry environments. Little is known regarding fluid production as a trait, and the selective forces thereof. Several *Nepenthes* produce a sticky, viscoelastic fluid, which has a benefit in increasing prey retention (Gaume and Forterre, 2007; Bonhomme *et al.*, 2011*b*; Bazile *et al.*, 2015; Kang *et al.*, 2021), and is even more effective on flies than on ants (Bazile *et al.*, 2015). Even watery fluid can have a retention function due to surface tension-lowering compounds that can be plant- or microbially produced, as observed in Sarraceniaceae (Juniper *et al.*, 1989; Armitage, 2016b). Plant-produced viscoelastic fluid composed of polysaccharides may have relatively high metabolic costs similarly to waxes made of long-chain polycarbonates. This is supported by the strong negative correlation observed in *Nepenthes* species between wax density and fluid viscoelasticity (Bonhomme *et al.*, 2011*b*). Again, this suggests the existence of a tradeoff in the investment of one pitcher trait involved in prey retention over another, the outcome of which may be related to sunlight levels, and/or temperature and humidity, and/or the type of insects predominantly present in the environment. Bonhomme *et al.* (2011b) observed that the species with the most viscoelastic fluids were most often species growing in mountains, where ants are rarer than in lowlands and flies are more abundant in relative proportions.

Many pitcher plants synthesize a complex cocktail of digestive enzymes that are secreted into the fluid (Saganová *et al.*, 2018; Adamec *et al.*, 2021). Evidence suggests that the proteins present in the digestive fluid of some pitcher plants may be species-specific and could correlate with trapped items (Biteau *et al.*, 2013; Rottloff *et al.*, 2016; Saganová *et al.*, 2018). Inducible enzyme production may reduce the metabolic costs relative to constitutive production (Pavlovič and Saganová, 2015). Furthermore, Rottloff *et al.* (2016) speculated that even though a particular enzyme is present in the secretome of pitcher plants, fluid pH may influence their activity, possibly rendering them inactive. The exact metabolic costs of the compounds secreted by the pitcher are under-explored, but some species appear to ‘outsource’ their digestive function to mutualistic microbes or animals they interact with (Lam *et al.*, 2017, 2018*a*, 2019*b*; Schӧner *et al.*, 2017), presumably offsetting those costs. *Nepenthes ampullaria* and *N. bicalcarata*, for example, may save energy (probably in the form of ATP; see An *et al.*, 2001) by not acidifying the fluid environment to the same extent as other species in favour of outsourcing digestion (Moran *et al.*, 2010; Lam *et al.*, 2019*a*; Lam and Tan, 2020; Freund *et al.*, 2022; Gilbert *et al.*, 2022). Microbes in particular can take over the functions of enzyme production (Sickel *et al.*, 2016; Kocáb *et al.*, 2021) or even provide a short-cut to nutrient acquisition by fixing atmospheric nitrogen (Prankevicius and Cameron, 1991). Microbial outsourcing of digestive functions has also been found in non-pitcher CPs, with the recent discovery of key enzyme-producing fungi in sundews (Sun *et al.*, 2014); N-fixing bacteria are also known from bladderworts (Sirová *et al.*, 2014). Examining the physiological costs involved in the production of pitcher fluid compounds, the active acidification and overall regulation of the fluid environment, as well as the consequent assimilation of nitrogen and other nutrients would present a fuller picture of the payoffs involved in carnivory investment.

**Fig. 2.**
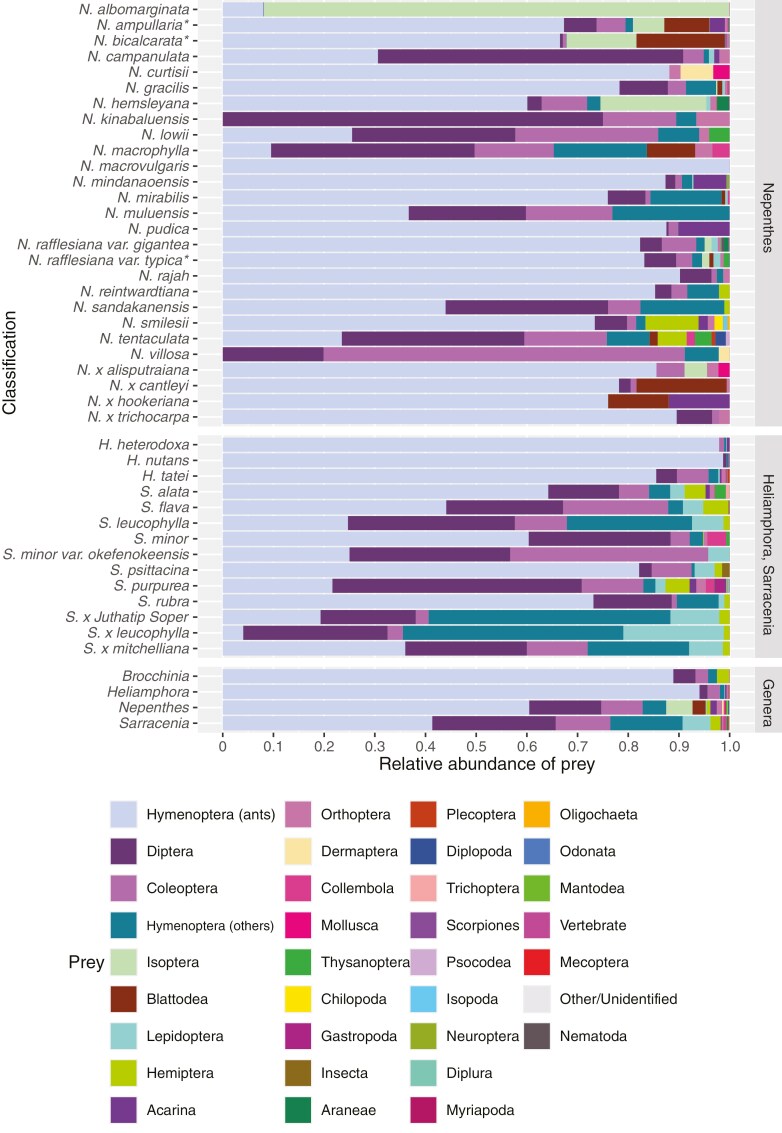
Relative prey abundance of *Nepenthes*, *Brocchinia*^†^, *Heliamphora* and *Sarracenia* at the species and genus level. *Prey capture includes unquantified vegetation. ^†^*Brocchinia reducta* was the sole species of its genus included in the analysis and therefore was not included in the cross-species comparison.

The quantity and identity of prey (‘diet’ or ‘prey spectrum’) can also be considered as a trait or rather the consequence of a constellation of pitcher traits, and thus may also vary among and within species. Considering that carnivory is the primary function of pitchers, there are surprisingly few data on prey capture by pitcher plants. Overall, data on prey capture only exist for *Sarracenia*, *Heliamphora*, *Brocchinia* and *Nepenthes*, with no documented data from *Darlingtonia*, *Cephalotus* or *Catopsis berteroniana* (Fig. 2). For most studies, taxonomic identification is only to the level of order, and collections made in a single day. This misses variation at finer taxonomic resolutions and variation in prey capture over time, and probably skews prey capture data toward less digestible prey (e.g. beetles and ants). The prey spectra among these pitcher plant lineages are broadly similar, comprising mainly ants (Formicidae), but the quantity and taxonomic composition vary considerably within and between taxonomic groups of pitcher plants (Kato *et al.*, 1993; Moran, 1996; Adam, 1997; Rembold *et al.*, 2010). This prey capture has been demonstrated to contribute 10–80 % of the total nitrogen content of pitchers/plants (Ellison and Gotelli, 2001). For *Sarracenia*, total prey capture has been quantified for 11 species and hybrids at 43 sites in total, and populations at different sites vary in the amount and composition of prey (Fig. 2; Supplementary Data). For example, the number of prey items in *Sarracenia purpurea* pitchers varied by 20 times between six sites in Britain and Ireland, where the species has naturalized (Whatmore *et al.*, 2022). In two neighbouring *Darlingtonia* populations in Plumas Co., California, average prey biomass in pitchers collected at shaded sampling sites was approximately half that of pitchers collected in sunny habitats, and was positively associated with pitcher leaf size in both habitats (Armitage, 2017).

In *Nepenthes*, prey spectra can differ amongst sympatric species (Chin *et al.*, 2014; Gaume *et al.*, 2016). A reasonable hypothesis is that divergence in the genus may be driven by nutrient competition (Pavlovič, 2012; Thorogood *et al.*, 2018), though direct empirical tests investigating competition are rare (but see Lam *et al.*, 2018*b*). Several species in Borneo have overlapping distributional ranges and have evolved specializations linked to nutrient sources including particular insect groups (Moran *et al.*, 2001), mammalian faecal capture (Clarke *et al.*, 2009; Greenwood *et al.*, 2011; Schӧner *et al.*, 2017) and leaf litter (Moran *et al.*, 2003; Pavlovič *et al.*, 2011). This diversity in pitcher function appears to be the result of an adaptive radiation driven by dietary shifts, analogous to well-known examples in animals, such as the diverse beak shapes of Darwin’s finches (Thorogood *et al.*, 2018). However, the trapping mechanisms of most of the ~200 documented species have never been observed. Recent work has used a mathematical modelling approach to provide a theoretical basis for how prey capture may be influenced both by peristome shape and relative size (Moulton *et al.*, 2023). This work suggests prey capture success is linked to geometric complexities, and hints at a fine-tuning of peristome size to optimize capture likelihood for a given shape and size. For example, species such as *N. veitchii* produce a conspicuously broad, oblique peristome. The model predicts that optimal levels of peristome flaring are consistent with those observed in nature, suggesting that these features confer a selective advantage in the capture-versus-construction tradeoff. *Nepenthes veitchii* often forms a distinct tree-climbing habit such that a portion of the peristome touches the vertical axis of the supporting tree. A flared peristome may act as a prey shuttle into the pitfall trap. By contrast, the model predicts that for the production of peristome ‘teeth’ (prominent spine-like, parallel features in, for example, *N. macrophylla*, *N. diabolica*, *N. villosa* and *N. hamata*), the cost significantly outweighs the benefit. Therefore, these structures – which have evolved independently – probably serve an unidentified function, for instance prey retention. This potential function could be determined by considering the prominent ‘fangs’ of *N. bicalcarata*, which have been shown to contribute to prey capture (Merbach *et al.*, 1999; Bonhomme *et al.*, 2011a; Thornham *et al.*, 2012); other species with flanges terminating in enlarged toothy projections may function similarly. In *N. hamata*, the highly ordered aquaplaning-promoting microtopography may be restricted to the flanges and teeth, with less ordered microtopography in flatter areas (U. Bauer, pers. obs.).

Despite some empirical data on the relationship between pitcher morphology and prey spectra in the genus *Nepenthes*, a similar level of detail is lacking for the other pitcher plant taxa, which also display a striking range of variation in pitcher morphology and physiology. For instance, in *Sarracenia*, the size and position of the lid varies from being reflexed away from the pitcher mouth (e.g. *S. purpurea*) to entirely obscuring its entrance (e.g. *S. minor*). Whatmore *et al.* (2022) found that pitchers with wider mouths caught more prey, while Cresswell (1993) also found that larger *Sarracenia purpurea* pitchers caught more prey. Dupont *et al.* (2023) showed that both pitcher size and quantity of VOCs are correlated with quantity and diversity of prey. Together, these studies show there are complex interactions between pitcher form and prey spectrum. Climatic and other environmental conditions as well as the faunal composition in particular habitats may also drive functional constraints. In considering current data on pitcher plant prey spectra, one notable dimension is the degree to which different taxa specialize on ants. At the genus level, *Nepenthes* specialize more on ants on average (~60 % of diet) compared to *Sarracenia* (~40 % of diet comprising ants), and both are dwarfed by *Brocchinia* and *Heliamphora* that both have upwards of 90–95 % ant prey (Fig. 2). Within *Nepenthes*, with the exception of the termite-specialist *N. albomarginata*, the species that do not have ants as the majority of their diet tend to be species from higher elevations such as montane *N. kinabaluensis*, *N. lowii*, *N. macrophylla*, *N. muluensis*, *N. sandakanensis*, *N. tentaculata* and *N. villosa* (Fig. 2). Cliff-dwelling *N. campanulata* also has <50 % ant relative abundance. Well-known consequences of elevational gradients on ant abundance could partly explain the general reduction of ants in the diets of high-elevation species (Szewczyk and McCain, 2016). However, more data from understudied pitcher plant lineages are needed to understand the apparent high degree of ant-specialization in the tepui-dwelling *Heliamphora* and *Brocchinia* species.

## BUDGETARY CONSTRAINTS ON PHOTOSYNTHETIC FUNCTION

Contrary to the common misconception that CPs are fully heterotrophic, relying solely on nutrients obtained from animal prey, all CPs conduct photosynthesis as their primary source of energy and carbon. In fact, CPs allocate a significant portion of the resources derived from photosynthesis towards trap construction and associated carnivorous functions, such as extrafloral nectar production and digestive enzyme secretion, generating a return on investment from carnivory. In pitcher plants, the photosynthetic cost of functional pitchers might be much higher than that of other CPs with simpler traps, such as sticky glandular traps, because of expensive pitcher construction and maintenance costs along with the photosynthetic inefficiency associated with their tubular shape. This suggests that pitcher plants must delicately balance the tradeoff between photosynthesis and carnivory. Few studies have directly compared the nutrient stoichiometry of carnivorous and non-carnivorous plants; Givnish and Shiba (2022) go further by examining these balances in tissues of both groups of plants co-occurring in the same bog. Pitchers (*Sarracenia*) and sundews (*Drosera*) were similarly nitrogen-limited, but the nutrient stoichiometry of aquatic bladderworts (*Utricularia*) was distinctly different. Overall, CPs had lower leaf C and N:P ratios and higher δ^15^N (partly due to prey capture) than non-CPs. More studies like this are needed to definitively parse the differences in nutrient and photosynthetic balance between pitchers and other carnivorous traps, and between CP and non-CP leaves generally; however, some interesting trends regarding pitcher budgetary constraints have already emerged from prior literature.

In optimal environments (sunny, moist and nutrient-poor) where photosynthesis is not limited, the tradeoff between photosynthesis and carnivory influences the ability of pitcher plants to enhance their overall fitness (i.e. through increased photosynthesis and relative growth rate) by obtaining additional nutrients from prey. However, in conditions or environments where the investment in carnivory outweighs the benefits, pitcher plants typically respond by reducing their allocation towards carnivory (e.g. reduction in trap size or quantity). A meta-analysis by Ellison (2006) indicated that increasing prey capture generally enhances growth for CPs, though this effect may be diminished in fertilized soils. In some instances, the plants might even completely forgo their carnivorous function. This diminishment of carnivorous function can take different forms, e.g. the production of flattened, non-carnivorous pitchers for photosynthesis in *Sarracenia* (Ellison and Gotelli, 2002; Ellison *et al.*, 2004) or complete pitcher abortion as observed in *Nepenthes* (Bazile *et al.*, 2012). Further illustrating the interaction between nutrition and trap function, Pavlovič *et al.* (2009) demonstrated that supplemental feeding in an experimental context increased both net assimilation (*A*_N_) and pitcher size in *Nepenthes talangensis*. Hence, the tradeoff between photosynthesis and carnivory presents constraints which shape the evolution and ecology of pitcher plants.

In a manipulative field experiment on *Sarracenia alata*, Segala and Horner (2023) tested the combined effects of light availability and prey capture on pitcher morphology, including leaf mass and pitcher aperture diameter, with greater diameters being important for effective prey capture (Heard, 1998; Bhattarai and Horner, 2009). Shading led to a reduction in pitcher diameter, revealing that not only do unusually high nutrient levels (N) tip the payoff balance away from carnivory and towards photosynthesis, but reduced energy from light (C) can also do the same. Segala and Horner (2023) also found that the prevention of prey capture led to a trend of reduced pitcher height. Further, the interaction between shading and feeding treatments was significant; shading significantly reduced leaf mass in fed plants, while shading had no effect on leaf mass in unfed plants. This shows that a lack of nutrient availability may diminish the ability of the plants to respond to changes in light availability.

## BUDGETARY CONSTRAINTS ON CARNIVOROUS FUNCTION

The LES focuses on tradeoffs among traits for the optimal allocation of nutrients for harvesting of light and CO_2_ by a leaf, and these tradeoffs would be expected to apply to pitcher plants. Because they are also nutrient-harvesting organs, the principles of the root economics spectrum may also play a role: that there is a tradeoff between nutrient exploration and resource conservation. Understanding how the strategies for managing the tradeoff between photosynthesis and prey capture are played out in different ecological situations provides useful insights into pitcher plant evolution and ecology. These may also constrain the reproductive ecology of pitcher plants, potentially limiting the suite of strategies that would be successful. Very little is known in this area; nevertheless, traps often seem to fall somewhere on the LES between low investment/short life span and high investment/durable. Construction costs seem to play a role, but there is not just one economically viable solution. Larger traps tend to be more sturdy, long-lived and costly (i.e. reinforced with lignin). This might be due to the demand to be able to withstand higher forces, both from fluid mass inside the trap, higher wind drag and also potentially larger prey. Lignin and other structural polysaccharides may in fact not be costly to CPs that grow in wet sunny habitats and are limited by N and P, not by photosynthesis. Several studies have examined concentrations of nutrients including N, P, K and trace elements in the tissues of both pitchers and laminae in several species of *Nepenthes* (Osunkoya *et al.*, 2007, 2008; Brearley and Mansur, 2012; Van der Ent *et al.*, 2015; Brearley, 2021; Mansur *et al.*, 2021, 2022, 2024); such data can provide pivotal insight into understanding these budgetary considerations to pitcher functions when combined with data on prey capture. The potential outsourcing of digestive functions to animal and/or microbial symbionts should also be considered, as interactions that further modify the outcome of budgetary balancing.

## ENVIRONMENTAL CONSTRAINTS ON CARNIVORY TRAITS

A clade-wide study of *Nepenthes* (94 species included) by Moran *et al.* (2013) used ecological niche models to identify the relationship between bioclimatic covariates such as annual mean, minimum and maximum temperatures and precipitation with particular classes of pitcher traits (peristome size, wax presence/absence, viscoelastic fluid). They found that humidity and seasonality were strong predictors of trapping traits such that pitchers could be classified based on morphology as belonging to ‘wet’ or ‘dry’ syndromes, with those species bearing highly specialized pitchers being most associated with perhumid climates of equatorial Southeast Asia, possibly due either to increased selection against novel, ‘riskier’ traits at the clade’s distribution margins, or through increased hybridization among *Nepenthes* species in the more species-rich Malay Archipelago. In a phylogenetic comparative analysis across the genus (85 species included), Gilbert *et al.* (2018) also found that species with higher elevational distributions (‘highland’ species) tended to produce shorter laminae and shorter pitchers (both upper and lower), and had larger peristome ribs compared with lowland species, though there are many exceptions to these trends. In concert, these results suggest that interspecific differences in trapping traits may be partially attributable to climatic drivers but further experimental work is needed to separate population genetic and ecological contributions to intraspecific pitcher trait plasticity.

Another important study by Gaume *et al.* (2016) examined interspecific differences in the pitcher traits of six sympatric *Nepenthes* species and how these differences relate to prey capture. They found compelling evidence for prey partitioning in this system such that each species in the community had a pitcher phenotype specialized on a different subset of the local insect community. While it is tempting to attribute these differences to ecological character displacement driven by resource competition, without data on the strength of resource competition among pitcher plants with similar traits, the ecological and evolutionary processes structuring these patterns remain vague. Another important outcome of their study was to demonstrate the extent of intraspecific variation in morphological characters of the seven focal Bornean species. Here, much more variation was observed in *N. bicalcarata* and *N. rafflesiana* pitchers than in *N. ampullaria*, *N. albomarginata*, *N. hemsleyana* or *N. gracilis.* However, the extent of intraspecific pitcher variation (both within and among lower and upper pitchers) was not clearly associated with a broader spectrum of captured prey. Instead, they found more variation at the interspecific level; specifically, prey diversity was positively correlated with pitcher mouth diameter, production of sweet odour and fluid acidity, with *N. rafflesiana* var. *typica* and *N. r.* var. *gigantea* being the most generalist species.

In a Singapore field study of *N. gracilis*, which exhibits polymorphism in pitcher colour, Gilbert *et al.* (2018) found a strong negative relationship between canopy cover and red pigmentation. This correlation between pigmentation and light environment was also suggested to be relevant more to interactions with herbivores rather than prey or for photoprotection.

To our knowledge, no studies have conducted similar trait–environment analyses on wild populations of *Heliamphora* or *Cephalotus.* However, both genera can be found growing across marked environmental gradients. In the case of *Heliamphora*, species can be found at the base and tops of tepui mountains where climatic conditions are considerably different. Likewise, *Cephalotus follicularis* can be found growing in habitats from directly adjacent to the ocean to more inland habitats; these may differ in soil mineral characteristics and osmotic conditions. How these species’ traits respond to such gradients remains an open question.

It may be worth mentioning as well that the organisms that pitcher plant species interact with may also respond to environmental gradients, thus indirectly influencing pitcher traits. For instance, ants, a key prey resource, are known to diminish in abundance with increasing elevation (Szewczyk and McCain, 2016); also, eukaryotic microbes living in pitcher fluid were found to be responsive to elevational gradients as well, though bacteria appear to be less sensitive to the same external gradient (Gilbert *et al.*, 2020). In general, to understand environmental constraints on carnivory traits across pitcher plant taxa, a closer look at environmental gradients will be insightful for finding correlates of variation in pitcher traits – incorporating morphology, physiology, prey spectra and symbiont interactions.

## ENVIRONMENTAL CONSTRAINTS ON SPATIAL DISTRIBUTION

Plants’ phenotypic traits are shaped in part by their local environments. That is, it may be more advantageous to possess certain traits at one end of an ecological gradient than the other, and this can manifest as either intra- or interspecific trait variation. Such ecological gradients are ubiquitous in nature and span scales ranging from centimetres to thousands of kilometres. Pitcher plants growing over such gradients are challenged to balance the budgetary constraints imposed in the previous section alongside the additional constraints of specific ecological pressures arising from both the abiotic and biotic components of the local environment. Such phenotype–environment correlations have been documented both within and among species across the vascular plant phylogeny (Bruelheide *et al.*, 2018), yet information specific to pitcher plants’ morphological tradeoffs and variation over ecological gradients remains scarce. Most pitcher plant species are relegated to extremely small geographical ranges, and within these minute ranges, local populations tend to be further fragmented into infrequent patches of favourable habitat. Therefore, a reasonable a priori hypothesis might be that pitcher plants – owing to the constraints detailed in the previous sections – are prevented from expressing the levels of phenotypic plasticity or capacity for adaptive trait divergence that allow them to tolerate even moderate levels of environmental variation. If so, this has important consequences for pitcher plant species’ range dynamics and vulnerability to a non-stationary climate.

In this section, we will attempt to understand the determinants of pitcher plants’ local and regional distributions from the perspective of their morphological traits. To do so, we will first review the literature on phenotype–environment correlations in pitcher plants. We will then contrast pitcher plant taxa in terms of habitat breadth and geographical range size with the goal of identifying potential axes of phenotypic variation and tradeoffs giving rise to these distributional trends.

The North American pitcher plant *Sarracenia purpurea* shows marked phenotypic variation across its large geographical range (Schnell, 1979), and is probably the most well-studied pitcher plant species from the perspective of ecological trait variation. Factors implicated in pitcher leaf trait variation (primarily in size and colour) include geographical location (Whatmore *et al.*, 2022), light availability (Yoon *et al.*, 2019), prey/nutrient availability (Farnsworth and Ellison, 2008; Yoon *et al.*, 2019), nitrogen deposition (Ellison and Gotelli, 2002), substrate pH (Karberg and Gale, 2013), wetland habitat type (Bott *et al.*, 2008) and regional climate (Freedman *et al.*, 2021). To date, only one study has combined multiple environmental factors to determine the most relevant environmental axes driving phenotypic variation in the species (Ellison *et al.*, 2004). Here, the authors measured six morphological traits on *S. purpurea*/*S. rosea* pitcher leaves from 39 locations spanning the entire native range of this species complex, and regressed these traits against principal axes representing aggregated climatic or pore-water chemical variables. The authors detected weakly significant positive relationships between precipitation and pitcher size, shape and peristome thickness. Pitchers from warmer sites tended to be taller and thinner than those from colder, more northern sites, and pitcher keel size was marginally positively correlated with increased dissolved nutrients in pore water. Overall, the relationships between morphology and site characteristics – including climate – were relatively weak and much variation in pitcher morphology among sites remained unexplained. One further study assessed latitudinal variation in germination characteristics of *S. purpurea* which, while not directly related to prey trapping adaptations, showed no clear association with latitude (Ellison and Gotelli, 2001).

Studies on spatial variation in pitcher morphology have also been carried out in other Sarraceniaceae. Comparing the morphology of *Sarracenia alata* growing at three neighbouring sites in Louisiana, Green (2006) found significant differences in pitcher height, hood area and funnel diameter between populations growing in a depression bog and a hillside seepage bog. These differences were associated with increased prey biomass by the larger hillside pitchers. In *Darlingtonia californica*, pitcher size peaks occurred at intermediate elevations, with the smallest pitchers at the lowest and highest elevation sites (Ellison and Farnsworth, 2005). While this comparison was only conducted on five populations ranging from 411 to 1241 m above sea level, anecdotal evidence from further *D. californica* populations ranging from sea level to 2000 m elevation generally accord with this trend (D. Armitage, pers. obs.). At high elevations, the small sizes of pitchers are probably due in part to exposed, windy environments and a short growing season. At sea level, while *D. californica* pitchers tend to be smaller, on average, than those at mid-elevations, there are also exceptions, possibly related to habitat quality, which is more variable among low-elevation populations. These size differences may also be genetic rather than environmental, given that one introduced population of *D. californica* in a sub-optimal coastal habitat in Mendocino Co., California, maintained the large stature of its source population from the Northern Sierra Nevada mountains.

Also, seed dispersal limitations may impose range restrictions on pitcher plants and prevent efficient habitat tracking. Seeds of *Sarracenia*, for example, travel an average of <10 cm (Ellison *et al.*, 2012), and while the hydrophobic seeds of Sarraceniaceae might have historically afforded them greater distance, the aquatic conditions required for such extension are more fragmented now than during their pre-Eocene heyday (Ellison, 2001). *Nepenthes* is dioecious, so colonization of both male and female seeds must establish in relatively close proximity in order for new populations to form (Baker, 1955). Germination data for *Cephalotus* suggest that recruitment occurs within a narrow range of environmental conditions (Just *et al.*, 2019), indicating that the window of opportunity is short and restricted. However, like many other functional traits, data on seed traits and dispersal in pitcher plants are relatively sparse in the literature.

## RELATIONSHIP BETWEEN ELEVATIONAL AND GEOGRAPHICAL RANGE IN *NEPENTHES*

Geographical ranges themselves (and the climate conditions they encompass) can also be considered traits of a species. Species in the Sarraceniaceae and Nepenthaceae tend to occupy small geographical ranges. Within each family, however, there are a small number of ‘outlier’ species with range sizes that are at least one order of magnitude larger than is typical for the family, for example *Sarracenia purpurea* in North America and *Nepenthes mirabilis* in Southeast Asia. These outliers may be well adapted to broader environmental conditions; however, this remains unexplored. Another possibility is that some species may have evolved in a time when their preferred niche was less fragmented such as the more ancestral lowland species *Nepenthes mirabilis* and *Nepenthes gracilis.* Many of the small-ranged *Nepenthes* species are endemic to one or a few isolated mountains, and although they sometimes occur there over relatively wide elevational gradients encompassing a range of climate zones, they do not occupy the lowland area that spans the space between mountain ranges. Some *Heliamphora* and many *Nepenthes* species only grow over exceedingly narrow climatic ranges. Highland species thus have greatly constrained niche space and may face greater loss of suitable habitat in the face of climate change (Schwallier *et al.*, 2016; Gray *et al.*, 2017).

While interspecific trait variation is one of the most apparent properties of pitcher plants, and evidence suggests that these traits at least partially covary with climate or other environmental factors, we can begin to assess whether traits of certain large-ranged ‘outlier’ species discussed above relate to their unique range sizes or environmental tolerances. Taking *Nepenthes* as an example, we can quantify the elevational range of each species as the difference between their highest and lowest observed distributions in the field (Moran *et al.*, 2013). Species with larger elevational ranges could generally be considered to be more tolerant of climate variation than those with very narrow elevational ranges. Since environmental tolerance is also a key determinant of geographical ranges (particularly over latitude), we can also rank *Nepenthes* species by their geographical range sizes, which might also positively covary with environmental breadth. To do so, we first pulled all georeferenced occurrence records of *Nepenthes* from various natural history databases (GBIF), and used them to fit species distribution models (SDMs) which correlate species presences with local climate variables and output a map of habitat suitability (Phillips *et al.*, 2006). By constraining these suitability maps to convex polygons of each species’ observed distribution, we can then estimate the overall geographical range sizes of each species (Kass *et al.*, 2022). While this is not a perfect way to estimate the range sizes of species with such limited numbers of occurrence records, it suffices for this illustrative comparative analysis. We asked whether the observed elevational distributions of *Nepenthes* species were associated with range sizes, with the expectation that taxa with wide elevational ranges should also have wide geographical ranges as well. Those species displaying the highest elevational and geographical ranges are anticipated to be few in number, but potentially possess some unique set of traits that might confer wide environmental tolerances.

**Fig. 3.**
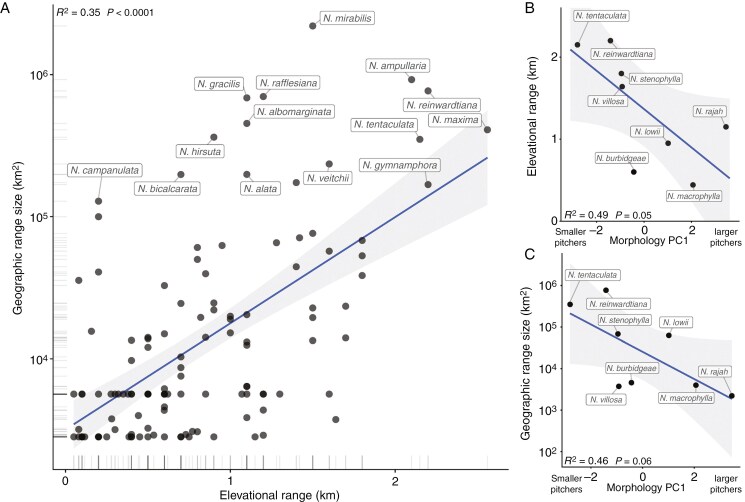
(A) Relationship between elevational range and geographical range size for *Nepenthes* species. Here, elevation limits were obtained from Clarke and Moran (2016) and range limits were estimated by fitting ecological niche models (when *n* ≥ 5) or 30-km-buffered convex hulls (when *n* < 5) to georeferenced occurrence data. B and C show the relationship between pitcher morphology and elevational/geographical range for sympatric *Nepenthes* species in Sabah (data from Chin *et al.*, 2010).

We found a significant, positive relationship between elevational and geographical range size (Fig. 3). Here, species such as *N. maxima*, *N. reinwardtiana*, *N. ampullaria*, *N. mirabilis* and *N. tentaculata* can be classified as having both the largest geographical ranges and larger than average elevational extents. What common traits, then, might these species share with one another? While there are no single, obvious similarities distinguishing these species from the rest, all do display remarkably high morphological variability in terms of growth form, pitcher coloration, pitcher size and peristome/operculum shape. However, these variants are typically relegated to very specific localities, rather than appearing repeatedly in similar environments on different landmasses, indicating they may not represent locally adapted ecotypes but rather arise as an evolutionary consequence of these species’ large, fragmented ranges.

Intraspecific trait variation arising from phenotypic plasticity is anticipated to be an advantageous trait for widely distributed species. Since few studies have simultaneously assessed intraspecific trait variation in different pitcher plant taxa (but see Chomicki *et al.*, 2024, who examined trapping-related traits in 40+ species), this prediction is difficult to assess. One study to do so measured the morphological traits of eight sympatric *Nepenthes* species on Mt Kinabalu and Mt Trusmadi in Sabah (Borneo) which included the small-ranged *N. burbidgeae*, *N. rajah*, *N. macrophylla* and *N. villosa* and larger-ranged *N. lowii*, *N. stenophylla*, *N. reinwardtiana* and *N. tentaculata* (Chin *et al.*, 2010). Re-analysis of the authors’ data reveals weakly negative trends between the first principal coordinate of pitcher morphology (including pitcher width, depth, height, volume and orifice depth and explaining 83 % of the variance), and both log geographical range size and elevational breadth (Fig. 3). The two species with the largest geographical/elevational ranges (*N. tentaculata* and *N. reinwardtiana*) in this study were also those with the smallest pitchers. There were, however, no relationships between range sizes and trait variation, as measured by the coefficient of variation. Further, for all morphological traits measured, interspecific variation was, on average, 2.5 times greater than intraspecific variation. This suggests that pitcher traits may be constrained more strongly by species-specific factors such as genetic background, rather than being free to converge on phenotypic optima in a common environment. Future work may take a macroecological perspective by integrating the frequency distributions of quantitative pitcher and leaf traits in *Nepenthes* to assess whether sympatric communities are non-random subsets of this distribution and to identify more morphological correlates of elevational and geographical range sizes.

## FUTURE DIRECTIONS: WHERE TO GO FROM HERE?

Similar to other plant organs, pitcher leaves of CPs show a broad trait spectrum (variation), both within and among independent lineages. The key to understanding the general ecological drivers and constraints to pitcher traits is leaf economy, which has been successfully explored and applied to other plant organs before and thus applies to pitchers as well. However, given that very little database trait data exist for pitcher plants, biologists must first focus on collecting trait data in a standardized and premeditated way. More specifically, the community must decide on which traits to collect, and by which methodology. Answers to these related questions, therefore, must engage with the observed or hypothesized tradeoffs either in accordance with, or contradicting, the LES. That is, to best understand developmental, functional, budgetary, or ecological constraints on pitcher leaves, which traits must we focus on collecting and comparing? This task and line of questioning has recently been posed for aquatic CPs as well (Bolpagni *et al.*, 2024).

Beyond the constraints discussed thus far, pitcher plants bring novel considerations, which can greatly expand our understanding of the factors shaping leaf construction writ large, whether carnivorous or not. One important insight is that leaves have multiple functions, not just photosynthesis. In the case of pitcher leaves, many key traits function in carnivory: attracting, trapping and digesting prey, and assimilating prey-derived nutrients. A perhaps underappreciated detail is that while roots are generally considered to be the assimilatory organs of non-CPs, leaves in general do have some capacity to uptake nutrients – even without specially adapted absorptive glands – such as via the stomata (Harrison *et al.*, 2000). The predominance of this absorptive function may exist as a gradient across all plants, with CPs and other specialized plants such as tank bromeliads at one end of the extreme. Incorporating this realization into a generalized economics spectrum model may not only improve our understanding of pitcher traits, but better contextualize variation in leaf traits overall.

Some insights may be more restricted to pitcher plants, such as traits that are most important to the trapping of prey. However, even these findings have broader implications for contemplating an expanded model of leaf trait evolution. Consider the springboard trapping mechanism; this is to our knowledge unique to pitcher plants and serves as an illustrative example of how complex traits (relying on multiple interdependent components for its function) can evolve from existing trait variation (Chomicki *et al.*, 2024). Additionally, springboard trapping raises a novel point, which is that certain traits may not be strictly dependent on material construction; rather, the same tissue arranged in different ways may achieve novel biomechanical properties. Much leaf trait work has framed tradeoffs and constraints in terms of construction costs, as we have also done here. However, leaves are not simply static objects, and mechanical properties may come into play even outside of carnivorous contexts as all leaves must deal with wind, rain and the movements of herbivores. Leaf biomechanics clearly matter in trapping strategies, but plants may also exploit the ways leaves are set in motion for the benefit of photosynthesis, e.g. shedding water to preserve photosynthetic efficiency. Thus, to fully understand global leaf trait variation space, simply measuring morphological and biochemical features in isolation may not suffice; more studies should investigate biomechanical features. All in all, many specific questions remain regarding pitcher traits (Box 3), but a lot of recent progress has been made and future directions have been identified. Ultimately, we believe it is possible to create an integrated model of leaf traits that can compare pitchers alongside ‘typical’ leaves; expanding the LES with these novel insights promises to greatly advance our knowledge even beyond this singular group of plants.

Box 1. **Comparison of different lineages of carnivorous pitcher plants**

The pitcher is one of a handful of divergent trapping structures that have evolved among carnivorous plants. In pitcher plants, the leaf has been modified into a tube-shaped pitfall trap. Insects attracted to the pitcher land on slippery rims which causes them to fall into a pool of digestive fluid. This strategy has evolved at least three times independently, in the families Sarraceniaceae (Ericales), Nepenthaceae (Caryophyllales) and Cephalotaceae (Oxalidales). In a broader sense, the pitfall strategy also applies to a handful of carnivorous bromeliad species (*Brocchinia reducta*, *B. hectioides*, *Catopsis berteroniana*) and the potentially carnivorous *Paepalanthus bromelioides* (Eriocaulaceae), also in the Poales. However, unlike in the case of pitcher plants *sensu stricto*, these pitfall traps consist of tanks created by multiple tightly overlapping leaves. In Sarraceniaceae, Nepenthaceae and Cephalotaceae, pitcher traps are all formed by individual leaves. These ‘true pitcher plants’ have many similarities in the general structure of their pitchers, including a lid covering the trap opening (the operculum), a slippery rim (the peristome), the tubular pitcher body with a slippery inner wall and the glandular digestive zone at the bottom of the inner pitcher wall, which also contains the digestive fluid.

The pitcher plants *sensu lato* in Bromeliaceae and Eriocaulaceae create fluid-filled tanks with multiple closely appressed leaves. By examining these multi-leaved pitcher plants, we can still test the extent to which the LES applies to carnivorous pitcher plants. While developmental constraints of single-leaved pitchers are probably already low, the developmental constraints of creating a tank bromeliad pitcher may be even lower, as it requires no rolled leaf. However, the functional requirement to attract, trap and digest prey, as well as to convert carbon to sugar, remains. Although each individual leaf does not function as a pitcher, the individual leaves are still governed by functional constraints on the entire pitcher’s ability to catch prey. For example, individual leaves produce a crumbling wax layer on the inner wall that interferes with adhesion between insect feet and the pitcher surface (Gaume *et al.*, 2004; Nishi *et al.*, 2012). In contrast to single-leaved pitchers, there is likely to be between-leaf variability in leaf photosynthetic efficiency; those forming the tank are quite vertical, reducing photosynthetic efficiency, but outer leaves are at a more typical angle for bromeliads and should have a more typical photosynthetic contribution to the plant. Thus, the net cost of each leaf is probably more variable in multi-leaved pitchers than in single-leaved species. Here we hypothesize similar functional constraints between single-leaved and multi-leaved pitchers, lower developmental constraints in multi-leaved pitchers, and budgetary constraints are more variable. Examining the various constraints on each leaf of a carnivorous bromeliad would test the limits of the LES.

Box 2. **Pitcher traits in large-scale plant trait databases**

Large-scale collaborative databasing efforts have potentiated a move toward trait-based ecology in the past two decades, which has provided new insights into ecosystem processes, trait evolution and plant function. These databases provide free access to trait data allowing for a diversity of large-scale studies that seek to describe and explain the vast trait variation found globally. One example of such a plant trait database is TRY (Kattge *et al.*, 2020), which is one particularly large and comprehensive database. As of the time of access, TRY contains 15 409 681 trait records from 305 594 species. This is a massive amount of data, but there are still gaps in our knowledge. One major gap concerns the tradeoffs involved in multi-function plant structures, such as those required for both carbon fixation and nutrient or water acquisition, for example the photosynthetic roots of epiphytic orchids or the prey trapping structures of carnivorous plants – the latter of which are the focus of this review.

To examine the state of trait data for these plants, we downloaded the data for all available pitcher plant species in the TRY database (Kattge *et al.*, 2020); this yielded 135 species with a total of 143 available traits. In contrast, if a random subset of 135 non-carnivorous species is selected, its trait table yields an average of 418 total measured traits (Supplementary Data). This illustrates that, even considering the relatively small number of species, pitcher plants have not been subject to the same depth and breadth of trait measurements, relative to non-carnivorous plants within this database. Compared to other plant groups, either fewer traits have been recorded and uploaded for pitcher plants, and/or researchers have yet to determine a standardized set of trait measurements that would facilitate ease of entry into large databases. Consequently, cross-species trait comparisons are less frequent within pitcher plants as a group. This is surprising, given that leaf and pitcher trap trait variation in these clades can be very high and are often used for species delimitation. This presents an opportunity for a more consistent trait-based approach to pitcher plant biology, expanding our database of traits to better-understand these evolutionary enigmas, and how they fit into the existing framework.

Box 3. **Assorted unanswered questions and future directions**

**Developmental**

The low developmental costs of transforming a flat leaf to a pitcher are understood, but less is known regarding the developmental forces shaping intraspecific variation:

- Nepenthaceae produce dimorphic pitchers – what developmental programming regulates the production of upper versus lower pitchers in *Nepenthes*?
- What controls the geometry of the trap shape, i.e. more cylindrical versus rounder pitcher shapes?
- What controls the angle of the lid and orientation of the pitcher aperture?
- Are there any developmental costs to pitcher fluid formation? The total volume of fluid appears to be under genetic control – consider the stable difference in fluid volume between *Nepenthes rafflesiana* and the closely related *N. hemsleyana*, the latter of which produces a lower volume.
- Are there any developmental constraints/costs to pigment pattern formation, including transparent windows found in *Darlingtonia* and *Sarracenia minor*?
- What constraints are there related to the expression of genes and proteins related to carnivory within pitchers and non-pitcher tissues?
- Phytohormones such as abscisic acid, ethylene and gibberellin are known to regulate leaf phenotypic plasticity in non-carnivorous plants. But do they also participate in the plasticity of carnivorous plants? If so, how might their broad, pleiotropic functions lead to developmental or functional constraints that shape their regulatory capacity?

**Functional**

- Research on the functional ecology of pitcher trapping mechanisms have thus far focused on adult pitchers, but many pitcher plants including *Sarracenia*, *Heliamphora* and *Nepenthes* have clear morphological differences in the juvenile pitchers produced by seedlings and earlier ontogenetic life stages of the plant. The size and shape of these juvenile pitchers may be suboptimal considering the functional constraints of the adult-form pitchers. Do juvenile pitchers function for prey capture? How do the plants overcome these potential functional costs?
- To what extent do pitcher traits vary, within and between species? How does this variability impact prey capture characteristics, and do certain traits develop at the expense of others?
- What factors go into the regulation of fluid production?

**Budgetary**

- How do carnivory–photosynthesis tradeoffs change during ontogeny?
- How does the carbon investment (LMA) of carnivorous pitcher plants compare to non-carnivorous leaves in both range (are most CPPs on the high LMA
  , high leaf longevity
  end of the LES) and variability?

**Ecological**

- What is the ecological niche breadth of particular pitcher traits (e.g. trap size, pigmentation, peristomal structure)?
- What are the environmental cues that regulate pitcher expression in *Nepenthes*? Do these cues vary across environments?
- Given that the trapping tissues of Cephalotaceae and Sarraceniaceae are also their centres of photosynthesis, they must be continuously produced by the plant. In contrast, *Nepenthes* can withhold pitcher production but still meet its carbon demands in suboptimal environments. Do these fundamental differences manifest as increased levels of trait–environment correlations in the trapping structures of the former groups relative to the latter?
- If trait–environment correlations in pitcher morphology (and leaf) morphology are rare (as they appear to be) then what aspect of a species’ phenotype permits it to have wider environmental tolerances or geographial distributions than small-range congeners?
- Similarly, does the spatial separation of trapping and photosynthetic tissues (e.g. in *Nepenthes*) permit a wider environmental distribution and more interspecific trap variation?

## SUPPLEMENTARY DATA

Supplementary data are available at *Annals of Botany* online and consist of the following. Combined supplementary data.

mcaf024_suppl_Supplementary

## Data Availability

Supplemental data and associated code will be made available on the Michigan State University Github repository (https://gitlab.msu.edu/gilbe334/pitcher-economic-spectrum). For the purpose of open access, the authors have applied a Creative Commons Attribution (CC BY) licence to any Author Accepted Manuscript version arising from this submission.
